# Household Transmission of SARS-CoV-2: A Prospective Longitudinal Study Showing Higher Viral Load and Increased Transmissibility of the Alpha Variant Compared to Previous Strains

**DOI:** 10.3390/microorganisms9112371

**Published:** 2021-11-17

**Authors:** Cathinka Halle Julin, Anna Hayman Robertson, Olav Hungnes, Gro Tunheim, Terese Bekkevold, Ida Laake, Idunn Forland Aune, Marit Fodnes Killengreen, Torunn Ramsem Strand, Rikard Rykkvin, Dagny Haug Dorenberg, Kathrine Stene-Johansen, Einar Sverre Berg, Johanna Eva Bodin, Fredrik Oftung, Anneke Steens, Lisbeth Meyer Næss

**Affiliations:** Division of Infection Control, Norwegian Institute of Public Health, P.O. Box 222 Skøyen, 0213 Oslo, Norway; CathinkaHalle.Julin@fhi.no (C.H.J.); anna.hayman.robertson@fhi.no (A.H.R.); Olav.Hungnes@fhi.no (O.H.); Gro.Tunheim@fhi.no (G.T.); Terese.Bekkevold@fhi.no (T.B.); Ida.Laake@fhi.no (I.L.); IdunnForland.Aune@fhi.no (I.F.A.); MaritFodnes.Killengreen@fhi.no (M.F.K.); TorunnRamsem.Strand@fhi.no (T.R.S.); Rikard.Rykkvin@fhi.no (R.R.); DagnyCathrineHaug.Dorenberg@fhi.no (D.H.D.); Kathrine.Stene-Johansen@fhi.no (K.S.-J.); EinarSverre.Berg@fhi.no (E.S.B.); JohannaEva.Bodin@fhi.no (J.E.B.); Fredrik.Oftung@fhi.no (F.O.); anneke.steens@rivm.nl (A.S.)

**Keywords:** household transmission, SARS-CoV-2, COVID-19, Alpha variant, B.1.1.7, secondary attack rate, SAR, viral load, ddPCR

## Abstract

We studied the secondary attack rate (SAR), risk factors, and precautionary practices of household transmission in a prospective, longitudinal study. We further compared transmission between the Alpha (B.1.1.7) variant and non-Variant of Concern (non-VOC) viruses. From May 2020 throughout April 2021, we recruited 70 confirmed COVID-19 cases with 146 household contacts. Participants donated biological samples eight times over 6 weeks and answered questionnaires. SARS-CoV-2 infection was detected by real-time RT-PCR. Whole genome sequencing and droplet digital PCR were used to establish virus variant and viral load. SARS-CoV-2 transmission occurred in 60% of the households, and the overall SAR for household contacts was 50%. The SAR was significantly higher for the Alpha variant (78%) compared with non-VOC viruses (43%) and was associated with a higher viral load. SAR was higher in household contacts aged ≥40 years (69%) than in younger contacts (40–47%), and for contacts of primary cases with loss of taste/smell. Children had lower viral loads and were more often asymptomatic than adults. Sleeping separately from the primary case reduced the risk of transmission. In conclusion, we found substantial household transmission, particularly for the Alpha variant. Precautionary practices seem to reduce SAR, but preventing household transmission may become difficult with more contagious variants, depending on vaccine use and effectiveness.

## 1. Introduction

SARS-CoV-2, the virus that causes the respiratory disease COVID-19, was first detected in China in 2019 and spread rapidly throughout the world [1]. In March 2020, the World Health Organization (WHO) declared COVID-19 a pandemic. Households have been one of the most important sites of transmission in Norway [2], as well as in other countries [3,4]. It is therefore important to identify risk factors for household transmission and effective precautionary practices to contain the epidemic. To this end, the WHO encouraged its member states to perform household studies. Moreover, secondary attack rate (SAR), defined as the probability of onward infection from a primary case to close contacts, provides an important measure of the transmissibility of SARS-CoV-2.

During the pandemic, various genetic variants have evolved from the original SARS-CoV-2 virus [5]. Some of these variants have spread rapidly throughout the world, such as the Alpha variant/Variant of Concern (VOC) 202012/01 (Pango lineage B.1.1.7), which rapidly outcompeted other SARS-CoV-2 lineages in the UK after its emergence in November 2020 [6,7]. This variant is well known for its increased transmissibility, which could be caused by increased viral load, in combination with other factors [5].

The majority of household transmission studies have described transmission of the SARS-CoV-2 variants dominating in the early phase of the pandemic or have not described the genetic variant(s). The first confirmed case of the Alpha variant in Norway was reported in December 2020, and from mid-February 2021 until July 2021 it was the dominant variant [2,8]. Even though increased transmissibility of the Alpha variant has been shown [9,10,11], knowledge is still sparse regarding how it affects the SAR in households. Moreover, it is not clear whether the Alpha variant is associated with a higher viral load, and if viral load influences the risk of transmission. There is also conflicting evidence about the viral dynamics in children versus adults [12,13,14].

We conducted a prospective longitudinal household study to investigate the SAR in Norwegian households, and to identify risk factors for transmission and preventative measures, using frequent testing and biological sampling, together with questionnaire data. Close follow-up and systematic data collection allowed for determination of the role of viral load in transmission. We used the droplet digital PCR (ddPCR) technique to quantify SARS-CoV-2 viral RNA due to its greater accuracy and precision compared to traditional quantitative PCR (rRT-PCR) [15,16]. Moreover, we compared the SAR for the Alpha variant with the SAR for other circulating variants in Norway during the study period.

## 2. Materials and Methods

### 2.1. Study Design and Study Population

**Figure 1:**
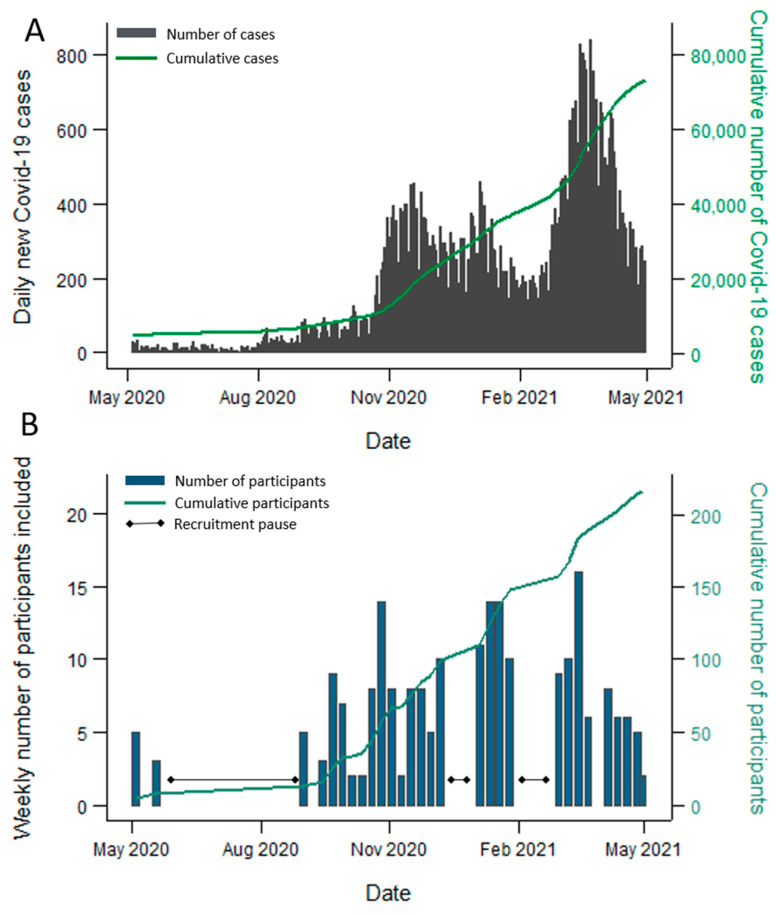
The course of the COVID-19 pandemic in Oslo/Viken, Norway, and the number of participants included in the study from 1 May 2020 to 30 April 2021. (**A**) The number of all reported laboratory confirmed COVID-19 cases in Oslo/Viken, Norway, during the study recruitment period. Participating households were recruited from these two counties. The daily number of cases is shown in grey, while the cumulative number of cases is shown in green. Source: Norwegian Surveillance System for Communicable Diseases (MSIS). (**B**) The number of included participants during the study recruitment period. The weekly number of participants included is shown in blue, while the cumulative number is shown in green. Three recruitment pauses are indicated.

The design of this prospective longitudinal study was based on the WHO Household Transmission Investigation protocol [17]. From May to June 2020, and from September 2020 to the end of April 2021 (excluding the last two weeks of December and the month of February), we recruited households of laboratory confirmed COVID-19 cases in the capital/county Oslo and the surrounding county Viken. The course of the pandemic in Oslo/Viken and of recruitment in this period are shown in Figure 1A,B, respectively. All households with a PCR-confirmed SARS-CoV-2 case aged ≥12 years, living with at least one other person aged ≥2 years, were eligible for participation. To avoid recruitment of households with co-primary cases, households with more than two members who tested positive on the same date were not eligible, unless the transmission dynamics were known. A further exclusion criterion was added when COVID-19 vaccines became available, whereby households with vaccinated individuals were not eligible. (Vaccines against SARS-CoV-2 only became broadly available towards the end of the recruitment period).

Primary cases and their household contacts were identified by the municipalities’ infection control teams following a positive SARS-CoV-2 PCR test and were subsequently contacted by the study team. Households willing to participate were visited at home, and written informed consent was obtained from the participants and/or their guardians before study inclusion.

The study was approved by the Regional Ethics Committee in Norway (#118354).

### 2.2. National COVID-19 Isolation and Quarantine Regulations

According to the Norwegian COVID-19 regulations, isolation was mandatory for persons with confirmed COVID-19. Isolation should be implemented at home or in similar accommodation for at least 8–10 days after symptom debut (recommendations varied throughout the study period), lasting at least three days after symptom relief. Asymptomatic cases had to isolate for 10 days after their initial positive PCR-test. In isolation, positive cases were instructed to stay ≥2 m from other household members, use separate bathrooms, towels, and bedrooms if possible. Household contacts were instructed to quarantine in their homes, maintaining an increased distance to other adults in the household.

### 2.3. Sampling and Data Collection

The first home visit for inclusion and sampling was termed Day 0, and seven further home visits for sampling were performed during the following 6 weeks (i.e., termed Day 3, Day 7, Day 10, Day 14, Day 21, Day 28, and Day 42) (Supplementary Figure S1).

Oropharyngeal (OP) samples and neat saliva samples were gathered from eligible participants on each visit to test for SARS-CoV-2 by rRT-PCR. Health care workers collected OP samples using OP flocked swabs (FLOQSwabs™Copan, Brescia, Italy), in 3 mL UTM (Universal Transport Medium, Copan, Brescia, Italy). Whole blood (Vacuette^®^EDTA-k2, Greiner Bio One, Kremsmünster, Austria) was collected once for each participant aged ≥18 years for blood typing. Saliva and blood for immunological analyses were also collected at Day 0, Day 7, Day 14, Day 28, Day 42, and Day 180 (outside the scope of this article).

All participants were asked to answer a questionnaire on Day 0 (Q-D0), to obtain information about the household in general, transmission risk factors, precautionary practices, clinical symptoms, and general health status. This questionnaire was adapted from the WHO protocol. The questions on behavioral risk factors in the Q-D0 related to the period up to 10 days prior to SARS-CoV-2 confirmation of the primary case, and precautionary practices after confirmation. An additional questionnaire (Q-DX) with questions on the suspected source of transmission, adherence to isolation/quarantine regulations, and a self-report of the severity of disease, was answered by participants at the home visit on Day 28/Day 42 or collected through phone interviews. In addition, a symptom diary adapted from the WHO protocol was completed daily from Day 0 to Day 28 by all participants.

### 2.4. Laboratory Testing

#### Detection of SARS-CoV-2 by rRT-PCR

All OP and saliva samples were tested for the presence of SARS-CoV-2 by rRT-PCR at the Norwegian Institute of Public Health (NIPH). RNA was extracted from samples (200 µL) using MagNaPure 96 DNA and Viral NA Small Volume kits (No. 6543588001, Roche, Basel, Switzerland), and eluted in 50 µL. Saliva samples with too much mucus were mixed 1:1 with sputum lysis buffer containing *N*-acetylcystein (10 g/L) and shaken for 30 min. Viral transport medium was added to saliva samples with insufficient volume before extraction. A semi-quantitative real-time reverse transcription polymerase chain reaction (rRT-PCR) was performed using the AgPath-ID One-step RT-PCR kit (No. 4387391, Life Technologies, Carlsbad, CA, USA) with primers and probes targeting two SARS-CoV-2 RdRp gene targets, developed at Institut Pasteur, Paris, France, and shared in the WHO protocol inventory [18]. A 25 µL reaction was set up, containing 5 µL of RNA. Criteria for a positive reaction were a cycle of threshold (Ct) value of less than 40 for both PCR targets and a credible amplification curve. Inconclusive results were resolved by repeating tests.

### 2.5. Quantitative Analysis of SARS-CoV-2 by Droplet Digital PCR (ddPCR)

For absolute quantification of viral load (SARS-CoV-2 RNA copies per µL eluate), ddPCR was performed on the saliva sample with the lowest Ct value for each participant, if sufficient material was available. The positive samples were further analyzed by droplet digital PCR (ddPCR) for absolute quantification. Results from the rRT-PCR analysis were evaluated to identify samples with high viral load that needed dilution to allow ddPCR quantification [19]. The PCR reagents for the 2019-nCoV CDC ddPCR Triplex Probe Assay were assembled according to the manufacturer’s instructions (Bio-Rad, Hercules, CA, USA). Subsequent water/oil emulsion formation, PCR thermal cycling and final droplet reading in the QX200 Droplet Digital PCR system (Bio-Rad, Hercules, CA, USA) were also done according to these instructions. The flow data were collected and initially analyzed by the QuantaSoft software (v1.7.4, Bio-Rad) that accompanied the droplet reader. Final analysis of the ddPCR data in the QuantaSoft Analysis Pro software (v1.0, Bio-Rad) revealed the number of SARS-CoV-2 RNA copies per µL eluate. The limit of detection of the ddPCR assay was 100 copies/mL according to the manufacturer’s instructions. For saliva samples that were initially diluted due to insufficient volume, the dilution factor was taken into account for the estimation of the number of SARS-CoV-2 RNA copies per µL eluate.

### 2.6. Sequencing of SARS-CoV-2

The positive samples were further analyzed by amplicon-based whole genome sequencing (WGS) of SARS-CoV-2 using the ARTIC-network nCoV-19 protocol v3 [20,21] using either the Nanopore or Illumina (MiSeq) technology at the NIPH; or the Swift Amplicon SARS-CoV-2 Panel (Swift Bioscience, Ann Arbor, USA) on Illumina (NovoSeq) at the Norwegian Sequencing Centre (NSC), according to the manufacturer’s instructions with minor modifications. The pipelines used to generate consensus sequences are publicly available on the NIPH and NSC Github sites [22,23]. The phylogenetic assignment of the consensus sequences was performed using Pangolin [24].

### 2.7. Blood Typing

Blood groups ABO and RhD were determined for all participants aged ≥18 years old using the Bio-Rad ID-Microtyping system at the blood bank of Oslo University Hospital.

### 2.8. Definition of Cases and Contacts

Household contacts were defined as individuals aged ≥2 years who resided with the primary case. A household contact was considered a secondary case if they had a positive PCR test (OP and/or saliva), and their symptom onset/PCR positive test (which ever came first; defined as T0) was within 14 days after T0 of the primary case. If a household contact had a T0 ≥2 days prior to T0 of the original primary case, the household contact was defined as an alternative primary case. If household members had the same T0, or ±1 day, they were re-defined as co-primary cases, unless the original primary case had a known source of infection outside of the household.

### 2.9. Definition of Variables

Household size was defined as the number of people living in the household. Overcrowding was defined as (1) the number of rooms in the property being less than the number of persons living in the household and (2) the number of square meters was less than 25 per person [25]. Symptom onset was defined as the date of presence of either cough, sore throat, runny nose, stuffy nose, dyspnea, fever, chills, change in taste/smell, headache, aches/pains, fatigue, nausea/vomiting, stomach pain/diarrhea, or a self-defined “date of symptom onset” (primary case only).

Clinical severity was based on the question: “How ill did you feel?” with the following 5 response categories: (1) “not ill”, (2) “not very ill”, (3) “moderately ill”, (4) “quite ill”, or (5) “seriously ill”, combined with the presence or absence of symptoms and/or dyspnea reported in the Day 0 questionnaire and symptom diaries. Dyspnea was considered a more severe symptom. Symptoms had to arise within 14 days after symptom onset, to ensure they were related to the SARS-CoV-2 infection. Severity was then defined according to response category and symptoms as follows: asymptomatic (category 1 or no reported symptoms), mild (categories 2–4 or symptoms without dyspnea), or moderate (category 5 or dyspnea).

Duration of detectable SARS-CoV-2 was defined as the mean number of days a household contact was positive for laboratory confirmed SARS-CoV-2, i.e., the date of the last positive saliva test minus the date of the first positive saliva test according to the test regime (sampling at Day 0, 3, 7, 10, 14, 21, 28, and 42). The date of the first positive sample was in some cases derived from the initial laboratory test taken through the municipality. Participants with any negative SARS-CoV-2 samples that had been diluted prior to rRT-PCR analysis were excluded from this analysis (*n* = 7; 4 children and 3 adults), resulting in a total of 56 household contacts in the analysis (21 children and 35 adults).

### 2.10. Study Samples Included in Analysis

**Figure 2:**
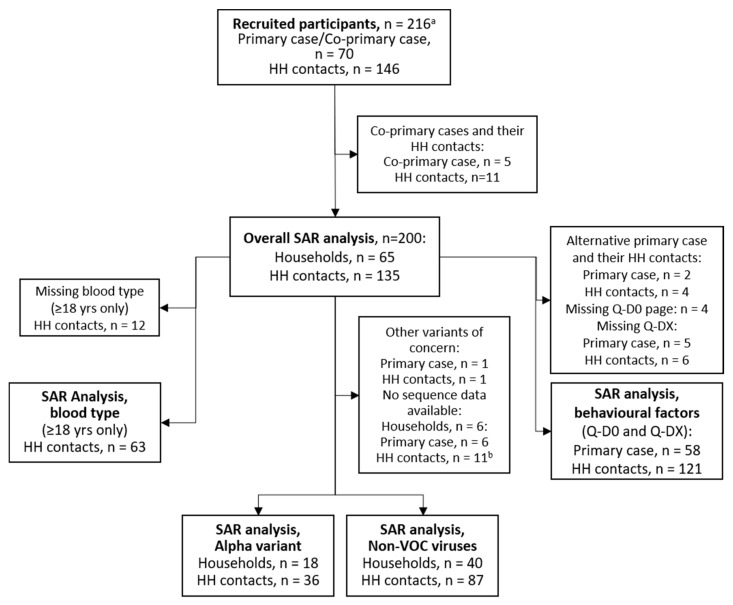
Flow chart of participant selection for the different analyses. ^a^ recruited participants were tested for SARS-CoV-2 by rRT-PCR and provided information on symptom onset (Q-D0 questionnaire). ^b^ includes household contacts that were SARS-CoV-2 negative.

For the main overall SAR analysis, households containing co-primary cases were excluded. Households with alternative primary cases were included in the overall SAR analysis but excluded from the analysis on behavioral factors and preventive measures due to lack of data from the Q-D0 questionnaire (Figure 2). For comparisons between genetic variants, households with the Alpha lineages were compared with non-VOC SARS-CoV-2 viruses, hereby referred to as non-VOC viruses [26], while households with other VOCs were excluded from the analyses (i.e., one household with the Beta variant). One household contact lacked variant data and was assigned the same variant as the primary case.

### 2.11. Data Analysis and Statistical Methods

The SAR was estimated as the proportion (%) of household contacts that were defined as confirmed cases [17]. Cluster robust standard errors were used to calculate 95% confidence intervals. The proportion of households with secondary transmission was also estimated. To test for differences in proportions, the Pearson chi-square test statistics was corrected with the second-order correction of Rao and Scott and converted into an F statistic [27].

To account for dependencies within households, a mixed-effect logistic regression model with a household-level random intercept was used to study the associations between potential risk factors for transmission and of infection among the household contacts. The multivariable models were adjusted for age and sex of the household contacts and of the primary cases, and household size. The analysis on associations between SARS-CoV-2 viral load (SARS-CoV-2 RNA copies/µL eluate measured by ddPCR) and symptoms was limited to the confirmed cases. For analyses with all cases, a mixed-effect logistic regression adjusted for age and sex was used, whereas for analyses only done on primary cases logistic regression was used. To study the association between genetic variant and viral load, a mixed-effect linear regression adjusted for age and sex was used. The mean duration of detectable SARS-CoV-2 was estimated for household contacts only, for children (<18 years) and adults (≥18 years), and cluster robust standard errors were used to calculate 95% confidence intervals. Primary cases were not included in this analysis as the majority were adults (due to the inclusion criteria of the study) and infection was likely detected later in the course of disease for these participants. To estimate the association between duration of detectable SARS-CoV-2 by rRT-PCR (in days) and age group, a mixed-effect linear regression was used.

All analyses were performed in STATA/SE 15.0 (StataCorp, College Station, TX, USA). A *p*-value of <0.05 was considered statistically significant (shown in bold in the tables).

## 3. Results

### 3.1. Baseline Characteristics of Households and Participants

We recruited 70 households, including 216 participants (Figure 2). Ninety eight percent of eligible household members agreed to participate in the study. Five cases were co-primary cases and were excluded together with their 11 household contacts. A total of 65 primary cases/households and their 135 household contacts (200 participants) were thus eligible for the evaluation of secondary transmission. Among the 65 households, 18 of the primary cases were infected with the Alpha variant, one with the Beta variant, and 40 with other circulating non-VOC viruses (Supplementary Table S1). Households with the Alpha variant were recruited between March and May 2021, while households with non-VOC viruses were mainly recruited before February 2021, reflecting the viral circulation in the study area during the recruitment period (Supplementary Figure S2). Sequence data showed the same genetic lineage for all sequenced members within individual households. Demographic and clinical characteristics of the participants in the SAR analyses are shown in Table 1. The median age of the participants was 31 years, and primary cases were generally older than household contacts (38 and 24 years, respectively). About 1/3rd of the participants were children aged <18 years, while only six were older than 65. The proportion of males and females was equal, and 51% were of Nordic ethnicity (but there was considerable missing data for this variable). A total of 16.5% of participants reported having chronic illnesses. Very few study participants were immunocompromised.

The median household size was four, ranging from two to six people, and families with young children constituted 43% of the households (Table 1). The household size was slightly smaller in households where participants were infected with the Alpha genetic variant (median = 3), compared with households infected with non-VOC viruses (median = 4). The remaining characteristics were broadly similar between these two groups.

Of the 200 participants, 132 (66%) were infected. Fourteen percent of the confirmed cases were asymptomatic, while 43% had mild disease, and 42% had a moderate disease, based on their reported symptoms within 14 days of their first positive PCR sample. Few study participants were hospitalized, and all were discharged the following day. There were slightly more asymptomatic cases (22%) among the Alpha variant participants compared with participants with non-VOC viruses (9%), although the difference was not significant (*p* = 0.09) (Supplementary Table S2). Severity also varied with age, with 36% of children (<18 years) being asymptomatic compared to adults (*p* < 0.01) (12% and 4% in those aged 18–39 and ≥40 years, respectively). Children were SARS-CoV-2 rRT-PCR positive for a shorter time period than adults (mean number of days 11.3 (95% CI 7.6–15.1) and 16.4 (95% CI 13.5–19.3), respectively, *p* = 0.03). The time period did not differ according to viral variant (data not shown). No association between blood type and variant was found (data not shown).

### 3.2. Secondary Transmission of COVID-19 in Households

Secondary transmission occurred in 60.0% of the households in the study (95% CI 47.4–71.4) (Table 2). The SAR among all household contacts was 49.6% (95% CI 37.8–61.5). Secondary transmission was significantly higher in households with the Alpha variant (83.3%, 95% CI 55.9–95.2) compared with non-VOC viruses (55.0% (95% CI 39.8–70.1), *p* = 0.04). For household contacts, SAR was 77.8% (95% CI 49.4–92.6) in households with the Alpha variant, compared with 42.5% (95% CI 28.7–57.7) in households with non-VOC viruses, resulting in a significantly higher adjusted odds ratio (OR) for secondary transmission in households with the Alpha variant (*p* = 0.03).

The median interval from the date of the first positive SARS-CoV-2 test (collected by the municipality for the primary case) and the Day 0 visit in the study was 3 days (IQR; 2–4 days). A large proportion of the secondary cases (38.5%) were already infected at Day 0, while 61.5% of the secondary cases were detected during study follow-up. The overall median serial interval (the number of days between symptom onset of the primary case and of a household contact) was estimated to 4 days (range 1–11, *n* = 50). The median serial interval was similar for the Alpha variant (4 days, range 2–11, *n* = 17) and non-VOC viruses (4 days, range 1–9 days, *n* = 31). The overall median interval between symptom onset of the primary case and the first rRT-PCR-positive test of a household contact was 3 days (range 1–12, *n* = 60), and this interval was similar for Alpha (3 days, range 1–11, *n* = 25) and non-VOC viruses (4 days, range 1–9 days, *n* = 33).

### 3.3. Effect of Host and Household Characteristics on Secondary Transmission

Neither age (12–39 years compared to ≥40 years) nor sex of the primary case appeared to have an impact on SAR (Table 3). Notably there were few primary cases under the age of 18, therefore it was not possible to study the effect of age on transmission for primary cases aged 12–18 years.

Secondary infection amongst children aged 2–17 years was similar for those aged 18–39 (SAR 47% and 40%, respectively), while household contacts aged ≥40 years were more likely to be infected (69%) (Table 3). The sex and blood type of the household contacts did not impact the infection risk. Household contacts living in overcrowded houses had a higher infection risk than those not living in overcrowded houses (SAR 90% and 52%, respectively), but the difference was not significant when adjusted for age, sex, and household size. However, the number of overcrowded households was small. Secondary transmission did not differ with household size or number of bathrooms in the household.

No difference in clinical severity was observed between the Alpha variant and other strains among the primary cases (Supplementary Table S3). However, both fever and loss of taste/smell were significantly more common in primary cases with the Alpha variant compared to others (Supplementary Table S3). In addition, the SAR was higher if these symptoms were present (Table 4). If the primary case reported loss of taste/smell, the SAR was 60% versus 27%, and there was a similar trend for fever (61% versus 39%). Dyspnea in the primary case did not appear to influence the SAR, nor clinical severity.

### 3.4. Role of Viral Load Measured by ddPCR

As expected, the correlation between viral load (SARS-CoV-2 RNA copies/µL eluate) determined by ddPCR and the rRT-PCR Ct-values was strong (r = −0.859, *p* < 0.001). There was a trend that higher viral load measured by ddPCR was associated with increased risk of secondary infection (adjusted OR 3.05 (95% CI 0.84–11.0), *p* = 0.089). Higher viral load was also associated with increased risk of loss of taste/smell (adjusted OR = 1.4 (95% CI 1.06–1.85), *p* = 0.02) (Supplementary Table S4). However, despite an OR larger than 1, this association was not significant when looking at the primary cases only, possibly because of the lower sample size. The remaining symptoms were not significantly associated with viral load (Supplementary Table S4).

**Figure 3:**
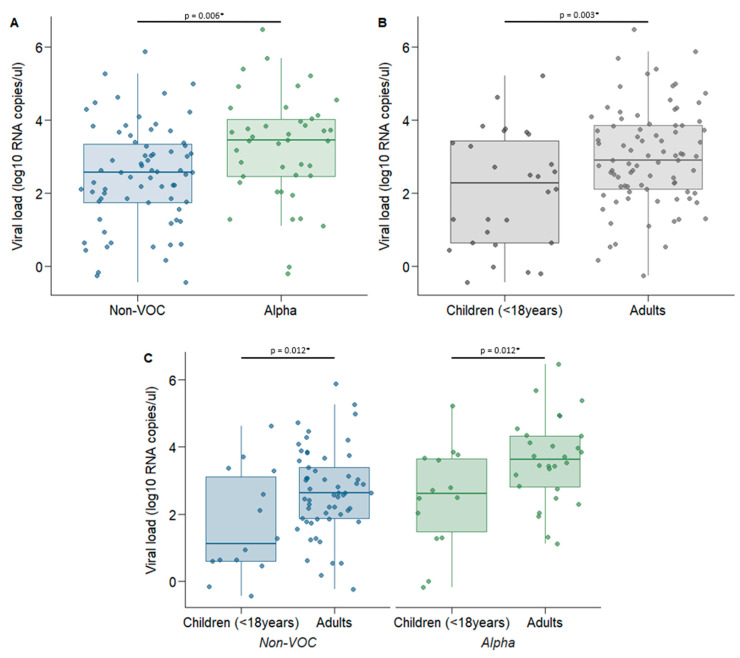
Comparison of viral load (log_10_ RNA copies/µL eluate) measured by ddPCR for genetic variant and age groups The saliva sample with the lowest Ct value for each participant was selected for quantification of viral load using ddPCR. (**A**) Comparison of viral load between non-VOC (*n* = 71) and Alpha viruses (*n* = 42), (**B**) Comparison of viral load between children (*n* = 28) and adults (*n* = 85) for all genetic variants combined, (**C**) Comparison of viral load for genetic variants and age groups: non-VOC; children (*n* = 14) versus adults (*n* = 57), and Alpha variant; children (*n* = 14) versus adults (*n* = 28). * *p* < 0.05. *p*-Values were estimated using a mixed-effects linear regression.

The viral load was significantly higher for the Alpha variant than for non-VOC viruses (mean 3.24 log_10_ and 2.48 log_10_ RNA copies/µL eluate, respectively, *p* = 0.006) (Figure 3A). We also found a significantly lower viral load in children than in adults (mean 2.09 log_10_ copies/µL RNA and 2.98 log_10_ copies/µL RNA, respectively) (Figure 3B), irrespective of virus variant (Figure 3C). The association between viral load and the Alpha variant remained significant in a mixed-effect linear regression model when adjusted for age and sex (adjusted regression coefficient of 0.87 (95% CI 0.34–1.40), *p* = 0.001).

### 3.5. The Impact of Behavioral Factors and Precautionary Practices on Secondary Transmission

None of the contact behaviors between the primary case and the household contacts prior to confirmation of infection of the primary case were significantly associated with SAR (Table 5). Nevertheless, there was a trend that the SAR was higher for contacts who shared a toilet, hugged, kissed, shook/held hands, slept in the same room, and shared a bed with the primary case before infection was confirmed.

After confirmation of the infection of the primary case, the only precautionary practice to significantly prevent household transmission was sleeping in a separate room from the primary case, with a SAR of 38%, compared to 67% for those who slept in the same room (*p* = 0.048) (Table 5). All other precautionary practices tended to lower the SAR, particularly isolation of the primary case, but associations were not statistically significant.

## 4. Discussion

This prospective longitudinal household study with close follow-up and systematic sampling shows a high overall SAR (49.6%), confirming that households are an important site of transmission. The SAR of the Alpha variant (B.1.1.7 VOC) was significantly higher, at 77.8%, compared with 42.5% for the other non-VOC viruses dominating in Norway until Feb/March 2021. A significantly higher viral load was found in the saliva of participants with the Alpha variant compared to the non-VOC viruses, which may contribute to the increased transmissibility. Close contact behavior prior to confirmation of infection of the primary case tended to give a higher SAR. However, we showed that SAR was reduced if the primary case slept in a separate room or was isolated from the rest of the household after infection was confirmed.

Our SAR-estimate of 42.5% for non-VOC viruses is higher than the household SAR found in other early reviews showing pooled SAR estimates around 17% [3,28]. However, our overall SAR of 49.6% is almost identical to the SAR of 49% found in a similar prospective household study from the USA performed during the same time-period [29] and in accordance with another Norwegian household study from the first wave of the pandemic, which estimated a SAR of 47% based on rRT-PCR and seroconversion [30]. Other studies performed in the UK, the Netherlands, and the US in the beginning/middle of 2020 also found similar SARs of 37–53% [31,32,33]. A more recent Norwegian national register-based study found a considerably lower household SAR of only 21% [34]. Register based studies are more sensitive to underreporting, as it is not mandatory to test all household members, which may in turn lead to an underestimation of SAR. In particular, parents may hesitate to test children because of discomfort with nasopharyngeal swabbing. Indeed, Fung et al. [28] showed that studies that tested household members more frequently observed higher SARs. In contrast to our study, none of the aforementioned studies sequenced positive virus samples or quantified viral load, and most were performed before the Alpha strain appeared.

The Alpha variant has been shown to be generally more transmissible than non-VOC viruses [10,11] and our study demonstrates this in a household setting. Our finding that SAR is significantly higher in households with the Alpha variant compared with non-VOC viruses, is in agreement with previous household studies [14,35,36]. However, we estimated a substantially higher SAR (78%) for the Alpha variant than was reported in these other studies (38%, 26%, and 42%), probably because they were registry based. In our study, the extensive testing at eight different time points over several weeks with both salivary and oropharyngeal samples, including testing of small children, probably enabled identification of most infected cases in the households, and thus contributed to our higher SAR estimates both overall and for the Alpha variant. We found no difference between the median serial interval for the Alpha variant and the non-VOC viruses, which is in accordance with other studies [37].

Previous estimates of SAR in children and different age groups, have been conflicting [3,29,33,38,39,40,41], probably due to various biases, as discussed by Goldstein and colleagues [41]. We found that the risk of transmission was similar for children (<18 years) and adults below 40 years, while household contacts aged ≥40 years had increased risk of secondary transmission. This is in contrast to the study by McLean et al., which found no difference of SAR in various age groups [29]. The age of the primary case was not associated with the risk of secondary transmission in the household. However, most of the primary cases in our study were >18 years old with few participants >65 years, therefore an effect of age of the primary case as found by McLean et al., could not be excluded. Although the SAR for children (<18 years) and adults under 40 years was similar, a larger proportion of children were asymptomatic. This suggests that children are equally susceptible to infection as younger adults, but present with milder symptoms.

We used ddPCR to accurately assess SARS-CoV-2 viral load and to avoid potential inference from inhibitory substances which may influence the results when using rRT-PCR for quantification [15]. Previous studies have had conflicting results regarding the relationship between viral variant and viral load [12,13,14]. Our results support that the Alpha variant is associated with a higher viral load. It has been argued that the time of sampling may obscure the comparison of viral loads between variants [12]. In our study, frequent sampling enabled the selection of the sample with the lowest Ct-value for the quantification of viral load by ddPCR, thus reducing the effect of timing of sampling collection. Furthermore, our finding was consistent when the analysis was limited to the household contacts only (data not shown), for whom sampling was performed earlier in the course of infection compared with the primary cases. A recent, prospective longitudinal cohort study from UK [42] found no difference in peak viral load with Alpha compared with pre-Alpha variants, but this could be due to methodological differences as self-swabbing of the upper respiratory tract only was used in their study and they did not test for viral load in saliva specimens. In addition, we measured viral load using a more accurate method (ddPCR).

We also demonstrated both lower viral load and shorter duration of viral detection in children compared with adults, which in accordance with some studies [43,44,45], while others have shown no difference in viral load [46]. Our results may suggest that it is more difficult to detect an active infection in children, and that the timing of the test is of importance. Further, loss of taste/smell in primary cases, a distinctive feature of COVID-19 infection [47], was associated with a significant increase in SAR, which may in part be explained by an increased viral load as observed in participants reporting loss of taste/smell. The association between taste/smell impairment and higher viral load has also been found by others [48,49]. This may be dependent on variant, as we found that loss of taste/smell was more common amongst primary cases with the Alpha variant.

Most contact behavior such as kissing, appeared to slightly increase the odds of secondary transmission, although not significantly. We found that sleeping separately from the primary case after confirmation of infection prevented secondary infection, as shown previously [40]. Other measures reducing contact with the primary case, especially isolation, also seemed to lower secondary transmission. This is in contrast to a similar household study by Miller and colleagues [38] that found no effect, possibly explained by transmission already occurring prior to laboratory confirmation of the primary case. Although we also observed that a high fraction of the transmission had occurred quite early, our findings still support the importance of starting precautionary practices after infection.

Vaccination campaigns against SARS-CoV-2 are anticipated to reduce household transmission. However, a recent study from the UK suggests that the vaccines may be less effective at reducing household transmission against currently circulating strains (e.g., the Delta variant) than expected [42]. It is possible that updated vaccines may alter this picture.

The present study has several limitations. First, our sample size was small, which limited the comparison between factors associated with the Alpha variant and other non-VOC viruses, such as symptoms and severity. Further, the study was not initially designed to evaluate differences in SAR between variants, and the dominance of the variants differed during the study period. We can therefore not exclude that climate, people’s behavior, or other factors, could have influenced our results. Quarantine and isolation guidelines were similar throughout the whole study period; thus, we assume that this has not significantly influenced our results. We cannot exclude that some participants may have used antiviral or antipyretic drugs during the study. However, antiviral drugs are not commonly used in Norway outside of hospital settings. Finally, the age span of participants was limited, with few elderly individuals and mostly adult primary cases.

## 5. Conclusions

In this prospective longitudinal household study, we found an overall SAR for household contacts of 50%. The SAR was considerably higher for the Alpha variant (78%) than for non-VOC viruses (43%). Furthermore, the viral load was higher for the Alpha variant which may explain its increased transmissibility. We also showed that age affects secondary infection, with higher SAR in household contacts older than 40 years. Loss of smell/taste in the primary case was associated with increased transmission. Implementation of precautionary measures after detection of the first SARS-CoV-2 case seems to reduce household transmission, in particular sleeping separately from the primary case. However, preventing transmission within a household will become increasingly difficult with the emergence of more contagious variants, depending on vaccine use and effectiveness. 

## Figures and Tables

**Table 1:** Demographic and clinical characteristics of all included participants and households, and Alpha variant and non-VOC virus households.

Participant Characteristics ^a^	All Participants	All Households (HH)		Alpha Variant Households		Non-VOC Virus Households	
	(N = 200)	Primary Cases (N = 65)	HH Contacts (N = 135)	Primary Cases (N = 18)	HH Contacts (N = 36)	Primary Cases (N = 40)	HH Contacts (N = 87)
**Age (years)**							
Median (range)	31 (2–73)	38 (2–71) ^b^	24 (2–73)	38 (2–68) ^b^	22 (2–63)	38 (15–68)	24 (2–70)
(IQR)	(14.5–42)	(31–45)	(8–38)	(28–43)	(8.5–38.5)	(30.5–48)	(9–40)
2–17, *n* (%)	63 (31.5)	3 (4.6)	60 (44.4)	2 (11.1)	16 (44.4)	1 (2.5)	39 (44.8)
≥18, *n* (%)	137 (68.8)	62 (95.4)	75 (55.6)	16 (88.9)	20 (55.6)	39 (97.5)	48 (55.2)
**Sex**							
Female, *n* (%)	104 (52)	35 (53.9)	69 (51.1)	10 (55.6)	16 (44.4)	19 (47.5)	49 (56.3)
Male, *n* (%)	96 (48)	30 (46.1)	66 (48.9)	8 (44.4)	20 (55.6)	21 (52.5)	38 (43.7)
**Ethnicity ^c^**							
Nordic, *n* (%)	101 (50.5)						
Part Nordic, *n* (%)	24 (12)						
Other, *n* (%)	7 (3.5)						
missing, *n* (%)	68 (34)						
**Chronic illness ^d^**							
yes, *n* (%)	33 (16.5)	13 (20.0)	20 (14.8)	4 (22.2)	4 (11.1)	7 (17.5)	14 (16.1)
no, *n* (%)	165 (82.5)	52 (80.0)	113 (83.7)	14 (77.8)	32 (88.9)	33 (82.5)	71 (81.6)
missing, *n* (%)	2 (1.0)		2 (1.5)		0 (0)		2 (2.3)
**Profession (age ≥ 16 yrs)**							
Healthcare, *n* (%)	28 (19.3)	13 (20.0)	15 (18.1)	9 (23.1)	10 (18.5)	3 (18.8)	3 (13.6)
Other, *n* (%)	117 (80.7)	49 (79.0)	68 (91.9)	30 (76.9)	44 (81.5)	13 (81.3)	19 (86.4)
**Household Characteristics**		**All Households**		**Alpha Variant Households**		**Non-VOC Virus Households**	
**Household size**							
2 persons, *n* (%)		24 (36.9)		6 (33.3)		15 (37.5)	
3 persons, *n* (%)		9 (13.9)		11 (61.1)		9 (22.5)	
4 persons, *n* (%)		22 (33.9)		1 (5.6)		7 (17.5)	
5–6 persons, *n* (%)		10 (15.3)		0 (0)		9 (22.5)	
**Young children ^e^**							
yes, *n* (%)		28 (43.1)		9 (50)		16 (40)	
no, *n* (%)		37 (56.9)		9 (50)		24 (60)	

^a^ the total number of households and household contacts in the Alpha variant vs. “non-VOC viruses” comparison does not add up to the “overall” total of 200, as some sequence data was lacking, and VOCs other than Alpha were excluded (Figure 2). ^b^ includes an alternative primary case, aged 2 years. **^c^** due to missing data, ethnicity was not stratified according to genetic variant. ^d^ cancer, diabetes, cardiovascular disease, high blood pressure, chronic lung disease, asthma, obesity, chronic liver disease, chronic hematological disorder, chronic kidney disease, chronic neurological impairment/disease, HIV, immunosuppressed, organ or bone marrow recipient. ^e^ households with minimum one child ≤12 years old.

**Table 2:** Comparison of transmission rates for all households and household contacts, and for Alpha variant versus non-VOC viruses.

Households ^a^ with Transmission	% with Transmission, 95% CI	PCR+ (*n*)/(N)	*p*-Value ^b^	Crude OR, 95% CI	*p*-Value		
All variants	60.0 (47.4–71.4)	39/65					
Non-VOC viruses	55.0 (39.8–70.1)	22/40		1 (Ref)			
Alpha variant	83.3 (55.9–95.2)	15/18	**0.04**	4.24 (0–4.2 × 10^32^)	**0.04**		
**Household Contact ^c^**	**SAR %,** **95% CI**	**PCR+** **(*n*)/(N)**	***p*-Value ^b^**	**Crude OR,** **95% CI**	***p*-Value**	**Adjusted OR ^d^,** **95% CI**	***p*-Value**
All variants	49.6 (37.8–61.5)	67/135					
Non-VOC viruses	42.5 (28.7–57.7)	37/87		1 (Ref)		1 (Ref)	
Alpha variant	77.8 (49.4–92.6)	28/36	**0.02**	65.7 (1.74–2481)	**0.02**	468 (1.8–1.2 × 10^5^)	**0.03**

^a^ including 65 households for all variants, and 18 households with Alpha variant, and 40 households with non-VOC viruses. ^b^ comparison of Alpha variant with non-VOC viruses. Pearson chi^2^ test statistics was corrected with the second-order correction of Rao and Scott and converted into an F statistic. ^c^ households with at least one confirmed case among its household contacts. ^d^ adjusted for the age and sex of the primary case and household contacts, and household size (number of persons per household). PCR+, PCR positive. *p*-Values < 0.05 are shown in bold.

**Table 3:** Secondary attack rates (SAR) and odds ratios (OR) for secondary infection of all household contacts (N = 135) according to characteristics of primary case, household contact characteristics, and household characteristics.

Characteristic	SAR %, (95% CI)	*p*-Value ^a^	PCR+ (*n*)/Total (N)	Crude OR (95% CI)	*p*-Value	Adjusted OR ^b^ (95% CI)	*p*-Value
	PRIMARY CASE CHARACTERISTICS						
**Age (yrs)**							
12–39 ^c^	47 (31–64)		33/70	1 (Ref)		1 (Ref)	
≥40	52 (35–69)	0.67	34/65	2.30 (0.25–21.1)	0.46	1.6 (0.15–16.9)	0.70
**Sex**							
Female	46 (30–63)		29/63	1 (Ref)		1 (Ref)	
Male	53 (35–70)	0.58	38/72	1.51 (0.18–12.7)	0.71	1.7 (0.15–18.7)	0.68
	HOUSEHOLD CONTACT CHARACTERISTICS						
**Age (yrs)**							
2–17 yr	47 (31–63)		28/60	1 (Ref)		1 (Ref)	
18–39	40 (25–56)		17/43	0.31 (0.05–2.04)	0.23	0.18 (0.02–1.33)	0.09
≥40	69 (49–83)	**0.03**	22/32	8.03 (1.15–56.2)	**0.04**	7.53 (1.07–52.8)	**0.04**
**Sex**							
Female	52 (38–66)		36/69	1 (Ref)		1 (Ref)	
Male	47 (32–62)	0.55	31/66	0.81 (0.22–3.01)	0.76	0.97 (0.24–3.91)	0.96
**Blood type (≥18 yrs)**							
O	48 (27–69)		10/21	1 (Ref)		1 (Ref)	
A	56 (37–73)		18/32	1.45 (0.38–5.5)	0.59	1.41 (0.40–4.96)	0.59
AB	33 (0–100)		1/3	0.49 (0.02–10.3)	0.64	0.40 (0.02–6.47)	0.52
B	71 (22–96)	0.61	5/7	3.03 (0.32–28.3)	0.33	3.02 (0.41–22.5)	0.28
	HOUSEHOLD CHARACTERISTICS						
**Household size**							
2 pers	54 (33–74)		13/24	1 (Ref)		1 (Ref)	
3 pers	59 (27–84)		10/17	1.72 (0.06–53.0)	0.76	2.0 (0.05–88)	0.71
4 pers	47 (28–68)		28/59	0.48 (0.03–7.72)	0.61	0.8 (0.04–17)	0.88
5–6 pers	46 (20–74)	0.87	16/35	0.48 (0.15–18.6)	0.68	0.7 (0.02–29)	0.84
**Overcrowding ^d^**							
No	52 (37–66)		47/91	1 (Ref)		1 (Ref)	
Yes	90 (24–100)	**0.01**	9/10	122.7 (0.16–94,464)	0.16	480.9 (0.11–2 × 10^6^)	0.15
**Number of bathrooms ^e^**							
1	58 (39–75)		36/62	1 (Ref)		1 (Ref)	
≥2	44 (27–61)	0.25	27/62	0.2 (0.02–2.7)	0.25	0.1 (0.01–2.2)	0.17

^a^ Pearson chi^2^ test statistics was corrected with the second-order correction of Rao and Scott and converted into an F statistic. ^b^ adjusted for the age and sex of the primary case and household contacts, and household size (number of persons per household), unless this was the factor being analyzed. ^c^ includes an alternative primary case, age 2. ^d^ data missing for 34 household contacts (25%). Overcrowding was defined as (1) the number of rooms in the property being less than the number of persons living in the household and (2) the number of square meters was less than 25 per person. ^e^ data missing for 21 household contacts (16%). PCR+, PCR positive. *p*-Values < 0.05 are shown in bold.

**Table 4:** Secondary attack rates (SAR) and odds ratios (OR) for secondary infection for all household contacts (N = 135) according to clinical severity and symptoms of primary case.

	SAR % (95% CI)	PCR+ (*n*)/ Total (N)	*p*-Value ^a^	Crude OR (95%CI)	*p*-Value	Adjusted ^b^ OR (95%CI)	*p*-Value
**Severity**							
Asymptomatic	33 (3–88)	4/12		1 (Ref)		1 (Ref)	
Mild	47 (27–68)	25/53		10.2 (0.17–613)	0.27	8.7 (0.13–594)	0.31
Moderate	54 (39–69)	38/70	0.54	12.1 (0.22–672)	0.22	11.8 (0.14–974)	0.28
**Loss of taste/smell**							
No	27 (13–47)	12/44		1 (Ref)		1 (Ref)	
Yes	60 (45–74)	55/91	**<0.01**	29.5 (1.33–654)	**0.03**	68.3 (1.95–2389)	**0.02**
**Fever**							
No	39 (24–57)	27/69		1 (Ref)		1 (Ref)	
Yes	61 (43–76)	40/66	0.08	10.3 (0.78–136)	0.08	10.4 (0.76–140)	0.08
**Cough**							
No	27 (11–53)	7/26		1 (Ref)		1 (Ref)	
Yes	55 (41–68)	60/109	**0.04**	10.0 (0.54–184)	0.12	10.2 (0.51–203)	0.13
**Dyspnea**							
No	46 (29–64)	31/68		1 (Ref)		1 (Ref)	
Yes	54 (38–69)	36/67	0.50	1.40 (0.17–12)	0.76	0.97 (0.1–9.51)	0.98

^a^ Pearson chi^2^ test statistics was corrected with the second-order correction of Rao and Scott and converted into an F statistic. ^b^ adjusted for the age and sex of the primary case and household contacts, and household size (number of persons per household). PCR+, PCR positive. *p*-Values < 0.05 are shown in bold.

**Table 5:** Effect of behavioral factors and precautionary practices on secondary attack rate (SAR).

		SAR% (95% CI)	PCR+/ HH Contacts	*p*-Value ^a^	Crude OR (95% CI)	*p*-Value	Adjusted OR ^b^ (95% CI)	*p*-Value
Behavioral factors: contact with the primary case *prior* to confirmation of infection								
Cared for	No	48 (35–61)	51/106		1 (Ref)		1 (Ref)	
	Yes	53 (27–78)	8/15	0.70	1.14 (0.31–2.74)	0.91	0.54 (0.04–6.79)	0.63
Hugged	No	39 (21–61)	11/28		1 (Ref)		1 (Ref)	
	Yes	52 (37–66)	48/93	0.28	3.26 (0.54–9.60)	0.20	3.90 (0.55–27.7)	0.17
Kissed	No	44 (29–60)	26/59		1 (Ref)		1 (Ref)	
	Yes	53 (36–70)	33/62	0.41	7.14 (0.86–9.10)	0.07	5.27 (0.63–44.0)	0.13
Shook/ held hands	No	47 (30–65)	16/34		1 (Ref)		1 (Ref)	
	Yes	49 (36–63)	43/87	0.80	3.51 (0.56–22.02)	0.18	4.30 (0.52–35.5)	0.18
Ate together	No	50 (25–75)	6/12		1 (Ref)		1 (Ref)	
	Yes	49 (36–62)	53/109	0.91	1.58 (0.20–2.78)	0.67	1.72 (0.19–15.1)	0.63
Shared a cup/glass/bottle	No	49 (36–62)	50/102		1 (Ref)		1 (Ref)	
	Yes	47 (20–77)	9/19	0.92	0.85 (0.10–0.25)	0.88	0.60 (0.06–5.64)	0.65
Slept in the same room	No	43 (26–61)	25/58		1 (Ref)		1 (Ref)	
	Yes	54 (39–68)	34/63	0.30	3.40 (0.79–4.66)	0.10	2.58 (0.53–12.5)	0.24
Shared a bed	No	42 (27–60)	25/59		1 (Ref)		1 (Ref)	
	Yes	55 (40–69)	34/62	0.19	2.93 (0.68–2.54)	0.15	2.18 (0.45–10.5)	0.34
Shared a toilet	No	20 (2–71)	2/10		1 (Ref)		1 (Ref)	
	Yes	51 (38–65)	57/111	0.10	117 (0.5–26,964)	0.085	125 (0.5–29,152)	0.08
Precautionary practices: performed by primary cases *after* confirmation of infection								
Isolated ^c^	No	67 (40–86)	33/22		1 (Ref)		1 (Ref)	
	Yes	42 (28–57)	37/88	0.10	0.07 (0.00–1.15)	0.06	0.06 (0.00–1.19)	0.07
Social distanced (≥2 m)	No	59 (40–76)	35/59		1 (Ref)		1 (Ref)	
	Yes	39 (23–57)	24/62	0.11	0.11 (0.01–1.31)	0.08	0.10 (0.01–1.32)	0.08
Used face mask	No	55 (38–70)	46/84		1 (Ref)		1 (Ref)	
	Yes	35 (19–56)	13/37	0.12	0.13 (0.01–1.62)	0.11	0.12 (0.01–1.51)	0.10
Slept in a different room	No	67 (44–85)	29/43		1 (Ref)		1 (Ref)	
	Yes	38 (25–54)	30/78	**0.03**	0.08 (0.01–0.98)	**0.048**	0.07 (0.00–0.98)	**0.048**
Used separate bathroom/toilet	No	53 (38–68)	41/77		1 (Ref)		1 (Ref)	
	Yes	41 (21–64)	18/44	0.35	0.30 (0.03–3.00)	0.31	0.30 (0.02–3.63)	0.34
Did not share a towel/items ^d^	No	69 (25–94)	11/16		NA		NA	
	Yes	49 (32–67)	30/61	0.34				

^a^ Pearson chi^2^ test statistics was corrected with the second-order correction of Rao and Scott and converted into an F statistic. ^b^ adjusted for age and sex of the primary case and household contacts, and household size (number of persons per household). ^c^ defined as resided in a separate room and kept ≥2 m distance from the rest of the household members, did not share a bedroom. ^d^ if shared a bathroom/toilet. PCR+, PCR positive. *p*-Values < 0.05 are shown in bold.

## Data Availability

The data presented in this study are available on request from the corresponding author. The data are not publicly available due to regulations in the Norwegian Health Research Act and the Norwegian Data Protection Act for use (and storage) of Personal Data related to health. Access to data can only be given to applicants with an ethical approval from their IRB or equivalent body, and an exemption from the duty of confidentiality from Health Registry controllers in Norway.

## Supplementary Materials

The following are available online at https://www.mdpi.com/article/10.3390/microorganisms9112371/s1, Figure S1: Flow chart of sampling of the participants at the different timepoints throughout the study. Figure S2: The proportion of genetic subgroups of all SARS-CoV-2 viruses analyzed by Next Generation Sequencing (NGS) from Oslo and Viken. Table S1: Frequency of participants with the various Pango lineages. Table S2: Comparison of clinical severity according to genetic variant (N = 123) or age (N = 132) amongst confirmed cases. Table S3: Comparison of clinical severity and symptoms according to genetic variant among primary cases only (N = 58). Table S4: Association between ddPCR log_10_ viral load (exposure) and symptoms (outcome).

## Abbreviations

HH contact	Household Contact
non-VOC	Non-Variant of Concern
VOC	Variant of Concern
HH	Households
IQR	Interquartile Range
SAR	Secondary Attack Rate
OR	Odds Ratio
CI	Confidence Interval

## References

[1] Hu B., Guo H., Zhou P., Shi Z.-L. (2021). Characteristics of SARS-CoV-2 and COVID-19. Nat. Rev. Microbiol..

[2] Norwegian Institute of Public Health (NIPH) COVID-19 Ukesrapport-uke 34. https://www.fhi.no/contentassets/8a971e7b0a3c4a06bdbf381ab52e6157/vedlegg/2021/ukerapport-uke-34-23.08---29.08.21.pdf.

[3] Madewell Z.J., Yang Y., Longini I.M., Halloran M.E., Dean N.E. (2020). Household Transmission of SARS-CoV-2: A Systematic Review and Meta-analysis. JAMA Netw. Open.

[4] Thompson H.A., Mousa A., Dighe A., Fu H., Arnedo-Pena A., Barrett P., Bellido-Blasco J., Bi Q., Caputi A., Chaw L. (2021). Severe Acute Respiratory Syndrome Coronavirus 2 (SARS-CoV-2) Setting-specific Transmission Rates: A Systematic Review and Meta-analysis. Clin. Infect. Dis..

[5] Oude Munnink B.B., Worp N., Nieuwenhuijse D.F., Sikkema R.S., Haagmans B., Fouchier R.A.M., Koopmans M. (2021). The next phase of SARS-CoV-2 surveillance: Real-time molecular epidemiology. Nat. Med..

[6] Rambaut A., Loman N., Pybus O., Barclay W., Barrett J., Carabelli A., Connor T., Peacock T., Robertson D., Volz E. (2020). Preliminary genomic characterisation of an emergent SARS-CoV-2 lineage in the UK defined by a novel set of spike mutations. Genom. Epidemiol..

[7] Chand M., Hopkins S., Dabrera G., Achison C., Barclay W., Ferguson N. (2020). Investigation of novel SARS-COV-2 variant Variant of Concern 202012/01. Public Health Engl..

[8] Norwegian Institute of Public Health (NIPH) COVID-19 Ukesrapport-uke 28. https://www.fhi.no/contentassets/8a971e7b0a3c4a06bdbf381ab52e6157/vedlegg/2021/ukerapport-uke-28-12.07---18.07.21.pdf.

[9] Piantham C., Linton N.M., Nishiura H., Ito K. (2021). Estimating the elevated transmissibility of the B.1.1.7 strain over previously circulating strains in England using GISAID sequence frequencies. medRxiv.

[10] Davies N.G., Abbott S., Barnard R.C., Jarvis C.I., Kucharski A.J., Munday J.D., Pearson C.A.B., Russell T.W., Tully D.C., Washburne A.D. (2021). Estimated transmissibility and impact of SARS-CoV-2 lineage B.1.1.7 in England. Science.

[11] Volz E., Mishra S., Chand M., Barrett J.C., Johnson R., Geidelberg L., Hinsley W.R., Laydon D.J., Dabrera G., O’Toole Á. (2021). Assessing transmissibility of SARS-CoV-2 lineage B.1.1.7 in England. Nature.

[12] Cosentino G., Bernard M., Ambroise J., Giannoli J.-M., Guedj J., Débarre F., Blanquart F. (2021). SARS-CoV-2 viral dynamics in infections with Alpha and Beta variants of concern in the French community. J. Infect..

[13] Kissler S.M., Fauver J.R., Mack C., Tai C.G., Breban M.I., Watkins A.E., Samant R.M., Anderson D.J., Ho D.D., Metti J. (2021). Densely sampled viral trajectories for SARS-CoV-2 variants alpha (B.1.1.7) and epsilon (B.1.429). medRxiv.

[14] Lyngse F.P., Mølbak K., Skov R.L., Christiansen L.E., Mortensen L.H., Albertsen M., Møller C.H., Krause T.G., Rasmussen M., Michaelsen T.Y. (2021). Increased Transmissibility of SARS-CoV-2 Lineage B.1.1.7 by Age and Viral Load: Evidence from Danish Households. medRxiv.

[15] Dyavar S.R., Ye Z., Byrareddy S.N., Scarsi K.K., Winchester L.C., Weinhold J.A., Fletcher C.V., Podany A.T. (2018). Normalization of cell associated antiretroviral drug concentrations with a novel RPP30 droplet digital PCR assay. Sci. Rep..

[16] Kim K.B., Choi H., Lee G.D., Lee J., Lee S., Kim Y., Cho S.Y., Lee D.G., Kim M. (2021). Analytical and Clinical Performance of Droplet Digital PCR in the Detection and Quantification of SARS-CoV-2. Mol. Diagn. Ther..

[17] World Health Organization (WHO) Household Transmission Investigation Protocol for 2019-Novel Coronavirus (COVID-19) Infection. https://www.who.int/publications/i/item/household-transmission-investigation-protocol-for-2019-novel-coronavirus-(2019-ncov)-infection.

[18] World Health Organization (WHO) Protocol: Real-Time RT-PCR Assays for the Detection of SARS-CoV-2 Institut Pasteur, Paris. Geneva: WHO. https://www.who.int/docs/default-source/coronaviruse/real-time-rt-pcr-assays-for-the-detection-of-sars-cov-2-institut-pasteur-paris.pdf?sfvrsn=3662fcb6_2.

[19] Basu A.S. (2017). Digital Assays Part I: Partitioning Statistics and Digital PCR. SLAS Technol. Transl. Life Sci. Innov..

[20] Quick J. nCoV-2019 Sequencing Protocol v3 (LoCost). Protocols.io. Version Created by Josh Quick. https://protocols.io/view/ncov-2019-sequencing-protocol-v3-locost-bh42j8ye.

[21] Tyson J.R., James P., Stoddart D., Sparks N., Wickenhagen A., Hall G., Choi J.H., Lapointe H., Kamelian K., Smith A.D. (2020). Improvements to the ARTIC multiplex PCR method for SARS-CoV-2 genome sequencing using nanopore. BioRxiv.

[22] Norwegian Institute of Public Health (NIPH) FHI Pipelines for SARS-CoV-2 Sequences Generated with the ARTIC Sequencing Protocol. https://github.com/folkehelseinstituttet/fhi-ncov-seq-pipelines.

[23] The Norwegian Sequencing Centre (NSC) SARS-CoV-2 Whole Genome Sequencing Based on Multiplexed Amplicon Method Using Short-Read Illumina Sequencers. https://github.com/nsc-norway/covid-seq.

[24] Rambaut A., Holmes E.C., O’Toole Á., Hill V., McCrone J.T., Ruis C., du Plessis L., Pybus O.G. (2020). A dynamic nomenclature proposal for SARS-CoV-2 lineages to assist genomic epidemiology. Nat. Microbiol..

[25] Statistics Norway Concept Variable-Crowded Dwelling. https://www.ssb.no/a/metadata/conceptvariable/vardok/3462/en.

[26] European Centre for Disease Prevention and Control (ECDC) SARS-CoV-2 Variants of Concern as of 24 June 2021. https://www.ecdc.europa.eu/en/covid-19/variants-concern.

[27] Rao J.N.K., Scott A.J. (1984). On Chi-Squared Tests for Multiway Contingency Tables with Cell Proportions Estimated from Survey Data. Ann. Stat..

[28] Fung H.F., Martinez L., Alarid-Escudero F., Salomon J.A., Studdert D.M., Andrews J.R., Goldhaber-Fiebert J.D., Group S.-C.M. (2020). The household secondary attack rate of SARS-CoV-2: A rapid review. Clin. Infect. Dis.

[29] McLean H.Q., Grijalva C.G., Hanson K.E., Zhu Y., Deyoe J.E., Meece J.K., Halasa N.B., Chappell J.D., Mellis A., Reed C. (2021). Household Transmission and Clinical Features of SARS-CoV-2 Infections by Age in 2 US Communities. medRxiv.

[30] Kuwelker K., Zhou F., Blomberg B., Lartey S., Brokstad K.A., Trieu M.C., Bansal A., Madsen A., Krammer F., Mohn K.G. (2021). Attack rates amongst household members of outpatients with confirmed COVID-19 in Bergen, Norway: A case-ascertained study. Lancet Reg. Health Eur..

[31] Bernal J.L., Panagiotopoulos N., Byers C., Garcia Vilaplana T., Boddington N., Zhang X.-S., Charlett A., Elgohari S., Coughlan L., Whillock R. (2020). Transmission dynamics of COVID-19 in household and community settings in the United Kingdom. medRxiv.

[32] Grijalva C.G., Rolfes M.A., Zhu Y., McLean H.Q., Hanson K.E., Belongia E.A., Halasa N.B., Kim A., Reed C., Fry A.M. (2020). Transmission of SARS-COV-2 Infections in Households-Tennessee and Wisconsin, April-September 2020. Morb. Mortal. Wkly. Rep..

[33] Reukers D.F.M., van Boven M., Meijer A., Rots N., Reusken C., Roof I., van Gageldonk-Lafeber A.B., van der Hoek W., van den Hof S. (2021). High infection secondary attack rates of SARS-CoV-2 in Dutch households revealed by dense sampling. Clin. Infect. Dis..

[34] Telle K., Jørgensen S.B., Hart R., Greve-Isdahl M., Kacelnik O. (2021). Secondary attack rates of COVID-19 in Norwegian families: A nation-wide register-based study. Eur. J. Epidemiol..

[35] Buchan S.A., Tibebu S., Daneman N., Whelan M., Vanniyasingam T., Murti M., Brown K.A. (2021). Increased household secondary attacks rates with Variant of Concern SARS-CoV-2 index cases. Clin. Infect. Dis..

[36] Lindstrøm J.C., Engebretsen S., Kristoffersen A.B., Rø G.Ø.I., Palomares A.D.-L., Engø-Monsen K., Madslien E.H., Forland F., Nygård K.M., Hagen F. (2021). Increased transmissibility of the alpha SARS-CoV-2 variant: Evidence from contact tracing data in Oslo, January to February 2021. Infect. Dis..

[37] Geismar C., Fragaszy E., Nguyen V., Fong W.L.E., Shrotri M., Beale S., Rogers A., Lampos V., Byrne T., Kovar J. (2021). Serial interval of COVID-19 and the effect of Variant B.1.1.7: Analyses from a prospective community cohort study (Virus Watch). medRxiv.

[38] Miller E., Waight P.A., Andrews N.J., McOwat K., Brown K.E., Katja H., Ijaz S., Letley L., Haskins D., Sinnathamby M. (2021). Transmission of SARS-CoV-2 in the household setting: A prospective cohort study in children and adults in England. J. Infect..

[39] Spielberger B.D., Goerne T., Geweniger A., Henneke P., Elling R. (2021). Intra-Household and Close-Contact SARS-CoV-2 Transmission among Children—A Systematic Review. Front. Pediatr..

[40] Ng O.T., Marimuthu K., Koh V., Pang J., Linn K.Z., Sun J., De Wang L., Chia W.N., Tiu C., Chan M. (2021). SARS-CoV-2 seroprevalence and transmission risk factors among high-risk close contacts: A retrospective cohort study. Lancet Infect. Dis..

[41] Goldstein E., Lipsitch M., Cevik M. (2021). On the Effect of Age on the Transmission of SARS-CoV-2 in Households, Schools, and the Community. J. Infect. Dis..

[42] Singanayagam A., Hakki S., Dunning J., Madon K.J., Crone M.A., Koycheva A., Derqui-Fernandez N., Barnett J.L., Whitfield M.G., Varro R. (2021). Community transmission and viral load kinetics of the SARS-CoV-2 delta (B.1.617.2) variant in vaccinated and unvaccinated individuals in the UK: A prospective, longitudinal, cohort study. Lancet Infect. Dis..

[43] Maltezou H.C., Vorou R., Papadima K., Kossyvakis A., Spanakis N., Gioula G., Exindari M., Metallidis S., Lourida A.N., Raftopoulos V. (2021). Transmission dynamics of SARS-CoV-2 within families with children in Greece: A study of 23 clusters. J. Med. Virol..

[44] Yan D., Zhang X., Chen C., Jiang D., Liu X., Zhou Y., Huang C., Zhou Y., Guan Z., Ding C. (2021). Characteristics of Viral Shedding Time in SARS-CoV-2 Infections: A Systematic Review and Meta-Analysis. Front. Public Health.

[45] Cendejas-Bueno E., Romero-Gómez M.P., Escosa-García L., Jiménez-Rodríguez S., Mingorance J., García-Rodríguez J. (2021). Lower nasopharyngeal viral loads in pediatric population. The missing piece to understand SARS-CoV-2 infection in children?. J. Infect..

[46] Madera S., Crawford E., Langelier C., Tran N.K., Thornborrow E., Miller S., DeRisi J.L. (2021). Nasopharyngeal SARS-CoV-2 viral loads in young children do not differ significantly from those in older children and adults. Sci. Rep..

[47] Tong J.Y., Wong A., Zhu D., Fastenberg J.H., Tham T. (2020). The Prevalence of Olfactory and Gustatory Dysfunction in COVID-19 Patients: A Systematic Review and Meta-analysis. Otolaryngol. Head Neck Surg..

[48] Jain A., Pandey A.K., Kaur J., Kumar L., Singh M., Das S., Purohit S. (2021). Is there a correlation between viral load and olfactory & taste dysfunction in COVID-19 patients?. Am. J. Otolaryngol..

[49] Nakagawara K., Masaki K., Uwamino Y., Kabata H., Uchida S., Uno S., Asakura T., Funakoshi T., Kanzaki S., Ishii M. (2020). Acute onset olfactory/taste disorders are associated with a high viral burden in mild or asymptomatic SARS-CoV-2 infections. Int. J. Infect. Dis..

