# Supplementary material for: Household Transmission of SARS-CoV-2: A Prospective Longitudinal Study Showing Higher Viral Load and Increased Transmissibility of the Alpha Variant Compared to Previous Strains
Source: Microorganisms. 2021 Nov 17;9(11):2371. doi: 10.3390/microorganisms9112371 (PMC8622435; doi:10.3390/microorganisms9112371)
Supplement: Supplementary file 1 [file microorganisms-09-02371-s001.zip › Supplementary_FigureS1.pdf]

	Collected from all participants (n=216)	*Collected only from participants aged ≥ 12 (n=166)*	
	**Saliva samples[a]**	**OP samples[a]**	**Blood samples**
**Day 0**	n= 198 (92%)	n= 156 (94%)	n= 151 (91%)
**Day 3**	n= 203 (94%)	n= 155 (93%)	
**Day 7**	n= 201 (93%)	n= 157 (95%)	n=147 (89%)
**Day 10**	n= 179 (83%)	n= 127(76%)	
**Day 14**	n= 191 (88%)	n= 147 (89%)	n=133 (80%)
**Day 21**	n= 170 (79%)	n= 123 (74%)	
**Day 28**	n= 160 (74%)	n= 127 (77%)	n=120 (72%)
**Day 42**	n= 156 (72%)	n= 113 (68%)	n=110 (66%)

Supplementary Figure S1: Flow chart of sampling of the participants at the different timepoints throughout the study. The percentages indicate how many of the eligible participants at each timepoint who provided the different samples. Abbreviations; OP; oropharyngeal.

[a] collected for viral detection by PCR.
