# Supplementary material for: Household Transmission of SARS-CoV-2: A Prospective Longitudinal Study Showing Higher Viral Load and Increased Transmissibility of the Alpha Variant Compared to Previous Strains
Source: Microorganisms. 2021 Nov 17;9(11):2371. doi: 10.3390/microorganisms9112371 (PMC8622435; doi:10.3390/microorganisms9112371)
Supplement: Supplementary file 1 [file microorganisms-09-02371-s001.zip › Supplementary_FigureS2.pdf]

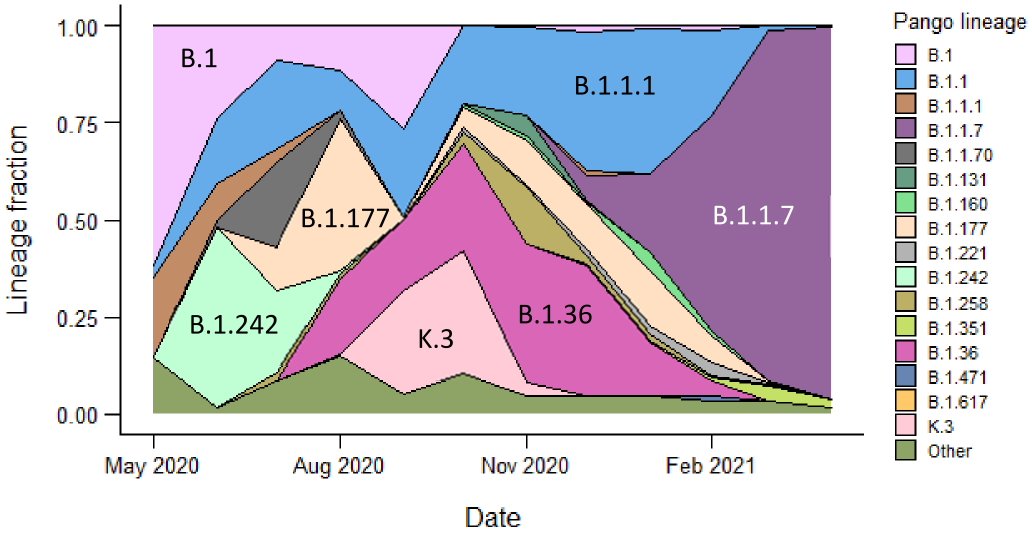

Supplementary Figure S2: The proportion of genetic subgroups of all SARS-CoV-2 viruses analyzed by Next Generation Sequencing (NGS) from Oslo and Viken, i.e. the counties of recruitment, per month during the study recruitment period, among sequences with >70% coverage. All subgroups with less than 5 occurrences are categorized as «Others», while «B» og «B.1» includes virus that were not allocated to a subgroup. Source: Surveillance data from the Norwegian Institute of Public Health (NIPH)
