# Supplementary material for: Household Transmission of SARS-CoV-2: A Prospective Longitudinal Study Showing Higher Viral Load and Increased Transmissibility of the Alpha Variant Compared to Previous Strains
Source: Microorganisms. 2021 Nov 17;9(11):2371. doi: 10.3390/microorganisms9112371 (PMC8622435; doi:10.3390/microorganisms9112371)
Supplement: Supplementary file 1 [file microorganisms-09-02371-s001.zip › Supplementary_TableS1.pdf]

Supplementary Table S1: Frequency of participants with the various Pango lineages.

Pango lineage	WHO label for VOCs[a]	Frequency
B.1.1.7	Alpha	46
B.1.351	Beta	2
B.1	-	2
B.1.1.1	-	3
B.1.1.141	-	1
B.1.1.151	-	1
B.1.1.153	-	1
B.1.1.162	-	1
B.1.1.277	-	6
B.1.1.333	-	2
B.1.1.39	-	1
B.1.1.64	-	8
B.1.36.21	-	16
B.1.160	-	6
B.1.177	-	10
B.1.258	-	1
B.1.367	-	7
B.1.398	-	1
K.3	-	7
Missing	NA	10

[a]VOCs; Variants Of Concern
