# Supplementary material for: Household Transmission of SARS-CoV-2: A Prospective Longitudinal Study Showing Higher Viral Load and Increased Transmissibility of the Alpha Variant Compared to Previous Strains
Source: Microorganisms. 2021 Nov 17;9(11):2371. doi: 10.3390/microorganisms9112371 (PMC8622435; doi:10.3390/microorganisms9112371)
Supplement: Supplementary file 1 [file microorganisms-09-02371-s001.zip › Supplementary_TableS2.pdf]

Supplementary Table S2: Comparison of clinical severity according to genetic variant (N=123) or age (N=132) amongst confirmed cases

Clinical severity	Genetic variant (n%[a])			Age (n%[a])			
	Alpha (N=46)	Non-VOC viruses (N=77)	p-value, chi^2	2-17 yrs (N=31)	≥18-39 yrs (N=52)	≥40 yrs (N=49)	p-value, chi^2
Asymptomatic	10 (21.7)	7 (9)		11 (35.5)	6 (11.5)	2 (4.1)	
Mild	16 (34.8)	38 (49.4)		17 (54.8)	16 (30.8)	24 (50.0)	
Moderate	20 (43.5)	32 (41.6)	0.09	3 (9.7)	30 (57.7)	23 (46.9)	**<0.00**

Abbreviations: non-VOC; non- Variant of Concern.

[a] proportion of cases (%)
