# Supplementary material for: Household Transmission of SARS-CoV-2: A Prospective Longitudinal Study Showing Higher Viral Load and Increased Transmissibility of the Alpha Variant Compared to Previous Strains
Source: Microorganisms. 2021 Nov 17;9(11):2371. doi: 10.3390/microorganisms9112371 (PMC8622435; doi:10.3390/microorganisms9112371)
Supplement: Supplementary file 1 [file microorganisms-09-02371-s001.zip › Supplementary_TableS3.pdf]

Supplementary Table S3: Comparison of clinical severity and symptoms according to genetic variant among primary cases only (N=58).

**Clinical symptom**	**Alpha n (%[a]) (N=18)**	**Non-VOC viruses n (%[a]) (N=40)**	**p-value (chi²)**
Severity			
Asymptomatic	2 (11.1)	3 (7.5)	
Mild	5 (27.8)	15 (37.5)	
Moderate	11 (61.1)	22 (55.0)	0.74
Cough	16 (88.9)	30 (75.0)	0.23
Fever	**15 (83.3)**	**18 (45.0)**	**0.01**
Dyspnea	10 (55.6)	21 (52.5)	0.83
Loss of taste/ smell	**16 (88.9)**	**23 (57.5)**	**0.02**

Abbreviations: non-VOC; non variant of concern

[a] proportion of cases (%)
