# Supplementary material for: Household Transmission of SARS-CoV-2: A Prospective Longitudinal Study Showing Higher Viral Load and Increased Transmissibility of the Alpha Variant Compared to Previous Strains
Source: Microorganisms. 2021 Nov 17;9(11):2371. doi: 10.3390/microorganisms9112371 (PMC8622435; doi:10.3390/microorganisms9112371)
Supplement: Supplementary file 1 [file microorganisms-09-02371-s001.zip › Supplementary_TableS4.pdf]

Supplementary Table S4: Association between ddPCR log_{10} viral load (exposure) and symptoms (outcome).

For all participants[a]	Crude OR (95% CI)	p-value	Adjusted[b] OR (95% CI)	p-value
**Symptom**				
Loss of smell/taste	**1.39 (1.06-1.82)**	**0.02**	**1.40 (1.06-1.85)**	**0.02**
Cough	**1.46 (1.01-2.11)**	**0.045**	1.37 (0.93-2.01)	0.11
Dyspnea	**1.36 (1.00-1.86)**	**0.048**	1.34 (0.96-1.86)	0.08
Fever	1.05 (0.82-1.35)	0.69	1.04 (0.8-1.34)	0.79
**For primary cases[c]**	**Crude OR (95% CI)**	**p-value**	**Adjusted[b] OR (95% CI)**	**p-value**
**Symptom**				
Loss of smell/taste	1.43 (0.89-2.28)	0.14	1.39 (0.85-2.28)	0.19
Cough	1.15 (0.69-1.9)	0.59	1.13 (0.68-1.89)	0.64
Dyspnea	1.05 (0.71-1.56)	0.80	1.06 (0.71-1.58)	0.78
Fever	1.2 (0.8-1.8)	0.38	1.2 (0.79-1.82)	0.39

Abbreviations: OR; Odds Ratio, CI; Confidence Interval

[a] n=116, mixed-effect logistic regression model with a household-level random intercept

[b] adjusted for sex and age

[c] n=56, logistic regression
